# Remote estimation of rapeseed yield with unmanned aerial vehicle (UAV) imaging and spectral mixture analysis

**DOI:** 10.1186/s13007-018-0338-z

**Published:** 2018-08-20

**Authors:** Yan Gong, Bo Duan, Shenghui Fang, Renshan Zhu, Xianting Wu, Yi Ma, Yi Peng

**Affiliations:** 1 School of Remote Sensing and Information Engineering, Wuhan University, Wuhan, 430079 China; 2 College of Life Sciences, Wuhan University, Wuhan, 430072 China; 3 Lab for Remote Sensing of Crop Phenotyping, Wuhan University, Wuhan, 430079 China

**Keywords:** Yield estimation, Rapeseed, Unmanned aerial vehicle, Canopy reflectance, Spectral mixture analysis, Abundance

## Abstract

**Background:**

The accurate quantification of yield in rapeseed is important for evaluating the supply of vegetable oil, especially at regional scales.

**Methods:**

This study developed an approach to estimate rapeseed yield with remotely sensed canopy spectra and abundance data by spectral mixture analysis. A six-band image of the studied rapeseed plots was obtained by an unmanned aerial vehicle (UAV) system during the rapeseed flowering stage. Several widely used vegetation indices (VIs) were calculated from canopy reflectance derived from the UAV image. And the plot-level abundance of flower, leaf and soil, indicating the fraction of different components within the plot, was retrieved based on spectral mixture analysis on the six-band image and endmember spectra collected in situ for different components.

**Results:**

The results showed that for all tested indices VI multiplied by leaf-related abundance closely related to rapeseed yield. The product of Normalized Difference Vegetation Index and short-stalk-leaf abundance was the most accurate for estimating yield in rapeseed under different nitrogen treatments with the estimation errors below 13%.

**Conclusion:**

This study gives an important indication that spectral mixture analysis needs to be considered when estimating yield by remotely sensed VI, especially for the image containing obviously spectral different components or for crops which have conspicuous flowers or fruits with significantly different spectra from their leave.

## Background

Rapeseed is an important cash crop cultivated primarily for its oil-rich seeds which can be processed into edible oil used all over the world. The byproducts of rapeseed are also widely used for animal feed, biofuel and medicine [1]. It is reported that in the last decade rapeseed displayed the highest production rise amongst oil crops [2] due to the long-term increase of global food and fuel demands. The accurate estimation of rapeseed yield, especially at regional scale, is of significance to evaluate the supply of vegetation oil and help enhance food security.

Remote sensing technique can efficiently obtain canopy spectra data from space, which carries valuable information indicating the canopy interaction with solar radiation such as vegetation absorption and scattering [3]. Many methods have been developed trying to relate the vegetation spectra to its optical properties for evaluating vegetation growth. Leaf pigments strongly absorb visible light thus reducing vegetation reflectance in the visible range [4], and vegetation reflectance in the near-infrared (NIR) range is affected by thick plant tissues and canopy structures [5]. Optical vegetation indices (VI), calculated from reflectance of different spectral ranges [6], have been developed to retrieve biophysical parameters such as leaf area index [7, 8], chlorophyll content [9, 10] and biomass [11, 12]. Instead of establishing regression algorithms of using VI to estimate vegetation parameter, machine-learning methods employ more sophisticated statistical techniques to develop relationships between the vegetation spectra and biophysical parameters [13]. For example, Bacour et al. [14] applied a neural network to estimate leaf area index and vegetation fraction with MERIS satellite reflectance at 11 bands. Verrelst et al. [15] used Gaussian process machine learning techniques to retrieve chlorophyll content with 62-band CHRIS satellite images. These methods can make full use of spectral information at all bands and be able to approximate complex non-linear functions, thus they often appeared more robust and adaptive than VI-based algorithms especially for hyperspectral data [16]. Despite spectral information, vegetation structure-related features can be also remotely estimated. Yue et al. [17] constructed crop 3D models using images taken from different positions for the same area, which clearly showed the height variations in wheat under different nitrogen and water treatments. Generally, VI-based methods are the mainstream approach for estimating biophysical parameters in various terrestrial ecosystems [18] and from various remote sensing platforms [19–21]. For multispectral data which is available for most current sensors, many experiments showed that machine-learning methods only slightly improved estimation accuracy compared with VI-based methods. The use of appropriate VI can give comparable performance to the complex machine-learning methods but with much more efficiency and feasibility [13].

The increase or decrease of crop photosynthesis capacity, which can be captured through spectral measures (e.g., VIs), directly affects plant development thus determining its ultimate yield. Thus VI showed the good potential as a basic and simple approach for remote estimation of crop yield at the large scale [22, 23]. Becker-Reshef et al. [24] found that in winter wheat the maximum Normalized Difference Vegetation Index (NDVI) derived from MODIS satellite data of each season closely followed the yield variations with the correlation coefficient above 0.74; Rahman et al. [25] utilized AVHRR-satellite-based NDVI and temperature data to model annual yield in rice with residual values in individual years around 4%; Sakamoto et al. [26] mapped U.S. corn yields successfully using Wide Dynamic Range Vegetation Index (WDRVI) derived from time-series MODIS data with the estimation error below 30% at the state level; Liang et al. [27] reported a good relationship between grape yield and NDVI derived from Landsat data having the correlation coefficient above 0.64. Remote sensing is able to offer the spatial and temporal information of the study site timely and economically. Their application for crop yield evaluation has been demonstrated across a wide range of scales and geographic locations [28–31].

Due to the limitation of the spatial resolution as well as the landscape fragmentation, there may be a considerable discrepancy between pixel sizes of the used remotely sensed images and much smaller sizes of the studied croplands. For example, MODIS satellite data, which is free available and widely used all over the world, obtains the daily global observations at the spatial resolution of 0.25–1 km. While the smallholder farms in China, which accounted to 98% of the total farm area in China, had the typical size smaller than 0.002 km^2^ [32, 33]. In this case, one pixel on an image encompasses several land cover types. Even for the high resolution data, the signal of one pixel can be contributed by multiple cropland components (e.g., soil, leaf, flower and fruit) that have significantly different spectra [34]. VI derived from spectra of such mixed pixels may include the data of components not or weakly related to yield, which introduces unexpected uncertainties for yield estimation. This problem is more obvious when applying to rapeseed. Unlike grain crops, conspicuous flowers will appear on top of the rapeseed canopy at its early reproductive stage and the flowering period may last more than 30 days [35]. Rapeseed flowers were bright yellow with dense petals that can scatter the radiation to all possible directions, while rapeseed leaves are green orienting nearly horizontal. With the same vegetation cover, it is observed that canopy spectra of rapeseed during flowering stage was twice as high as during green-up stage, especially in the green and NIR spectral ranges [36]. When the remotely detected canopy spectra is greatly mixed by flower and leaf spectra, the accuracy of estimating vegetation parameter with pixel-level VI would decrease. Behrens et al. [37] showed the weak correlations between NDVI and rapeseed biomass with the correlation coefficient below 0.1; Fang et al. [36] reported the uncertainties increased by 50% when using VI to estimate vegetation fraction in rapeseed during its flowering season. Canopy reflectance sensed from the space is confounded by different components of rapeseed cropland, and there is a need to consider the factor of spectral mixture that will influence the yield estimates especially during the flowering period.

Many studies used spectral mixture analysis to quantify the spectral contributions from different components within a pixel [38–40]. It assumes that the individual pixel is mixed by a few dominant components with different proportions that appear in the studied scene, and these components spectrally contribute to the total pixel signal at sub-pixel scale [41]. Endmembers, the dominant components of the image scene and not themselves mixed by other components, are firstly identified. A set of pure spectra of these endmembers is measured as field data, and the fraction of each endmember within a pixel can be estimated based on comparing the pixel spectra and field-collected endmembers’ spectra in multiple bands [42]. This method is commonly applied to assess vegetation properties. Based on measured spectra of two endmembers (bare soil and dense vegetation), Gitelson et al. [43] developed an approach to estimate vegetation fraction in sampling zones; Li and Strahler [44] proposed a model separating a pixel reflectance into reflectance of four components (sunlit ground, sunlit crown, shadowed ground and shadowed crown), and this model was further extended to estimate tree density in woodland using Landsat satellite data [45]. However, the analysis of how the spectral mixture will affect yield estimates and how to select appropriate endmembers for yield estimation in rapeseed has not been adequately elaborated and addressed.

Recently, Unmanned Aerial Vehicles (UAV) are increasingly used as an innovative remote sensing platform for environmental applications [46, 47]. Unlike field-collected data, UAV can fly over the predetermined area to obtain the images efficiently with very high spatial (e.g., centimeters) and temporal (e.g., daily observations) resolutions, which greatly reduces the labor and time costs [48]. In comparison to most satellite and airborne platforms, the availability of using customizable sensor on UAV as well as the flexibility of changing UAV flight altitude and attitude can give us an easy access to data with the spatial and spectral resolutions as required by users [49]. This is particularly beneficial for precision agriculture by offering the image with resolutions appropriately selected for detailed observations on the in-field crop growth. For example, Jin et al. [50] developed a method to estimate wheat density using images taken from a hexacopter flying at very low altitude (3–7 m); López-Granados et al. [51] mapped weed distributions in croplands based on images collected by UAV at different heights; Zhou et al. [52] predicted rice yield using multi-temporal images acquired by two cameras with different spectral ranges mounted on an UAV system. UAV-collected data is becoming a promising tool for monitoring crop growth and assisting in field managements.

This study explores to improve VI-based approach for estimating rapeseed yield by considering spectral mixture factors. The image of the study site was remotely obtained by an UAV system. The first objective is to compare and evaluate several widely used VIs for rapeseed yield estimation. The second objective is to identify and analyze the endmembers that appear in remotely sensed scene and mostly related to rapeseed yield. The final objective is to develop an approach for the accurate estimation of rapeseed yield with VI data and spectral mixture analysis.

## Methods

### Study area

**Fig. 1:**
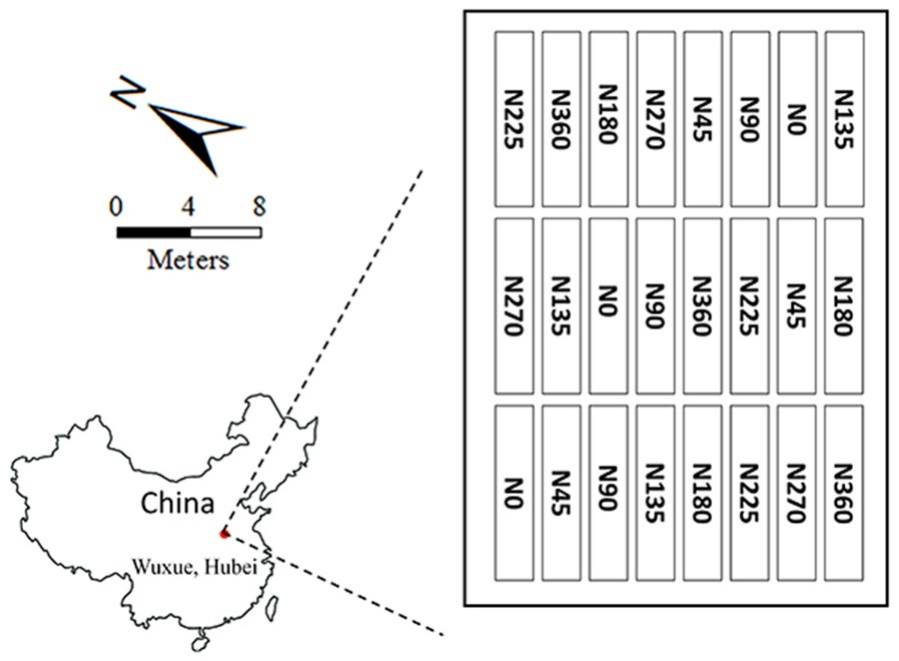
Study area in this study and the nitrogen fertilizer applications in 24 rapeseed plots

In this investigation, we studied 24 rapeseed plots located at Rapeseed Experiment and Research Base (30.1127°N,115.5894°E), Central China Agricultural University, Wuxue, Hubei, China. They were of the size about 15 m × 2 m and all planted with the same hybrid of rapeseed (Huayouza No.9) [53]. The field managements for these plots were similar except that different amounts of nitrogen fertilizer were applied. Eight nitrogen (N) rates (0, 45, 90, 135, 180, 225, 270 and 360 kg/ha) were utilized, and each rate was repeated on three randomly distributed plots (Fig. 1). All the plots were irrigated and weeded regularly. The growing season for our studied rapeseed was from Sept. 2014 to the following May. In this study, one UAV flight was arranged to obtain the image of study area on Mar. 21, 2015 during the early flowering stage of the rapeseed. In this period, rapeseed was on the stage that plants increase photosynthetic rates due to strong carbon sink of developing flowers and fruits [37]. Thus, the obtained image at this stage probably corresponded to the maximum photosynthesis capacity of rapeseed plants, which is indicative to its final yield. For all 24 plots, half of each plot was sampled periodically for crop growth evaluations while the other half of each plot was kept intact until the harvest date for yield determination.

### Rapeseed yield determination

The 24 rapeseed plots were harvested on 5 May, 2015. In each plot, half of the above-ground plant materials (around 15 m^2^) were all cut for yield determination. The harvested materials were exposed to the sun for 10 days for seed threshed. The seeds were then cleaned and put into an oven at 60 °C until their weight did not change (around 4 days). All the dry seeds were weighted together and the plot yield was calculated as the ratio of this total weight to the ground area (kg/ha). The final yield of 24 plots varied from 1000 to 3500 kg/ha, which represented a wide range of yield variation.

### Canopy reflectance and VI derived from UAV data

The UAV flight was carried out on Mar. 21, 2015 between 10:00 and 13:00 local time when changes in solar zenith angle were minimal and the weather was clear with low cloud cover observed. The Mini-MCA system (Mini-MCA 6, Tetracam Inc., Chatsworth, CA, USA) was mounted on an UAV (S1000, SZ DJI Technology Co., Ltd, Shenzhen, China) to obtain images of the studied area. Mini-MCA is consisted of six individual miniature digital cameras, and each camera lens was equipped with a customer-specified band pass filter centered at wavelength of 490, 550, 670, 720, 800 or 900 nm respectively at the band width of 10 nm. These bands were selected since they were commonly used for estimating vegetation photosynthesis-related parameters [37, 54, 55].

Prior to the flight, six cameras were co-registered in the laboratory using a camera distortion correction model [56] so that the corresponding pixels of each lens were spatial overlapped in the same focal plane. During the flight, a gimbal stable platform was used to help adjust the camera system pointing close to nadir [57], which minimized the fluctuations in collected reflectance due to variations of observation azimuth angles. The flight altitude was kept at 50 m above the ground to acquire images at the spatial resolution around 2.5 cm. For each exposure, six cameras simultaneously took a picture to produce a six-band-composite image of the study area.

In this study, the image digital numbers (DN) were converted to surface reflectance using the empirical line approach [58, 59]. Four calibration ground targets, providing a relatively flat response to incident radiation throughout the visible to NIR spectral ranges, were placed in the cameras’ field of view as a standard for image radiometric corrections. The calibration targets used in this study are made of highly durable woven polyester fabric at the size of 0.4 m × 0.6 m, having the relatively constant reflectance of 6%, 24%, 48% and 100%, respectively (more details can be found at: http://www.tetracam.com/Products_Ground_Calibration_Panels.htm). Assuming a linear relationship between surface reflectance and DN values, canopy surface reflectance $\uprho\left(\uplambda \right)$ can be calculated as [60, 61]:

1 $$\rho (\lambda ) = DN(\lambda ) \times G_{\lambda } + B_{\lambda } \quad (\uplambda = 490, 550, 670, 720, 800\; {\text{and}}\; 900\,{\text{nm}})$$

where ${\text{DN}}\left(\uplambda \right)$ is the digital number of a given pixel at wavelength $\uplambda$; ${\text{B}}_{\lambda }$ and ${\text{G}}_{\lambda }$ are bias and gains of the sensor at wavelength $\uplambda$. For each wavelength, B and G can be calculated based on DN values of pixels from four calibration targets (referring to $DN_{0.06}$, $DN_{0.24}$, $DN_{0.48}$, $DN_{1}$)

2 $$\left[ {\begin{array}{*{20}c} B \\ G \\ \end{array} } \right] = \left( {\left[ \begin{aligned} \begin{array}{*{20}c} 1 & {DN_{0.06} } \\ \end{array} \hfill \\ \begin{array}{*{20}c} 1 & {DN_{0.24} } \\ \end{array} \hfill \\ \begin{array}{*{20}c} 1 & {DN_{0.48} } \\ \end{array} \hfill \\ \begin{array}{*{20}c} 1 & {DN_{1.00} } \\ \end{array} \hfill \\ \end{aligned} \right]^{T} \left[ \begin{aligned} \begin{array}{*{20}c} 1 & {DN_{0.06} } \\ \end{array} \hfill \\ \begin{array}{*{20}c} 1 & {DN_{0.24} } \\ \end{array} \hfill \\ \begin{array}{*{20}c} 1 & {DN_{0.48} } \\ \end{array} \hfill \\ \begin{array}{*{20}c} 1 & {DN_{1.00} } \\ \end{array} \hfill \\ \end{aligned} \right]} \right)^{ - 1} \left[ \begin{aligned} \begin{array}{*{20}c} 1 & {DN_{0.06} } \\ \end{array} \hfill \\ \begin{array}{*{20}c} 1 & {DN_{0.24} } \\ \end{array} \hfill \\ \begin{array}{*{20}c} 1 & {DN_{0.48} } \\ \end{array} \hfill \\ \begin{array}{*{20}c} 1 & {DN_{1.00} } \\ \end{array} \hfill \\ \end{aligned} \right]^{T} \left[ {\begin{array}{*{20}c} {0.06} \\ {0.24} \\ {0.48} \\ {1.00} \\ \end{array} } \right]$$

Within each of 24 plots, we defined a maximum rectangle fitted the plot (including around 30,000 pixels). And the plot-level reflectance was calculated as the average value of all pixels within the defined rectangle. Plot-level VI was then retrieved from plot-level canopy reflectance (Table 1).

**Table 1:** Vegetation indices tested in this study

Vegetation indices	Formula	References
Normalized Difference Vegetation Index (NDVI)	$(\rho_{800} - \rho_{670} )/\left( {\rho_{800} + \rho_{670} } \right)$	Rouse et al. [62]
Red edge chlorophyll index (CI_rededge_)	$\rho_{800} /\rho_{720} - 1$	Gitelson et al. [63]
Green chlorophyll index (CI_green_)	$\rho_{800} /\rho_{550} - 1$	Gitelson et al. [63]
Visible Atmospherically Resistant Index (VARI)	$(\rho_{550} - \rho_{670} )/\left( {\rho_{550} + \rho_{670} } \right)$	Gitelson et al. [43]
Ratio Vegetation Index (RVI)	$\rho_{800} /\rho_{670}$	Jordan et al. [64]
Difference Vegetation Index (DVI)	$\rho_{800} - \rho_{670}$	Richardson et al. [65]
Renormalized difference Vegetation Index (RDVI)	$\sqrt {NDVI \times \left( {\rho_{800} - \rho_{670} } \right)/2}$	Roujean et al. [66]
Enhanced Vegetation Index (EVI)	$2.5\left( {\rho_{800} - \rho_{670} } \right)/\left( {\rho_{800} + 6\rho_{670} - 7.5\rho_{490} + 1} \right)$	Liu et al. [67]
Triangular Vegetation Index (TVI)	$0.5\left[ {120\left( {\rho_{800} - \rho_{550} } \right) - 200\left( {(\rho_{670} - \rho_{550} } \right)} \right]$	Broge et al. [68]
Soil Adjusted Vegetation Index (SAVI)	$\left( {1 + L} \right)(\rho_{800} - \rho_{670} )/\left( {\rho_{800} + \rho_{670} + L} \right)$	Huete [69]

### Spectral mixture analysis and endmember abundance

**Fig. 2:**
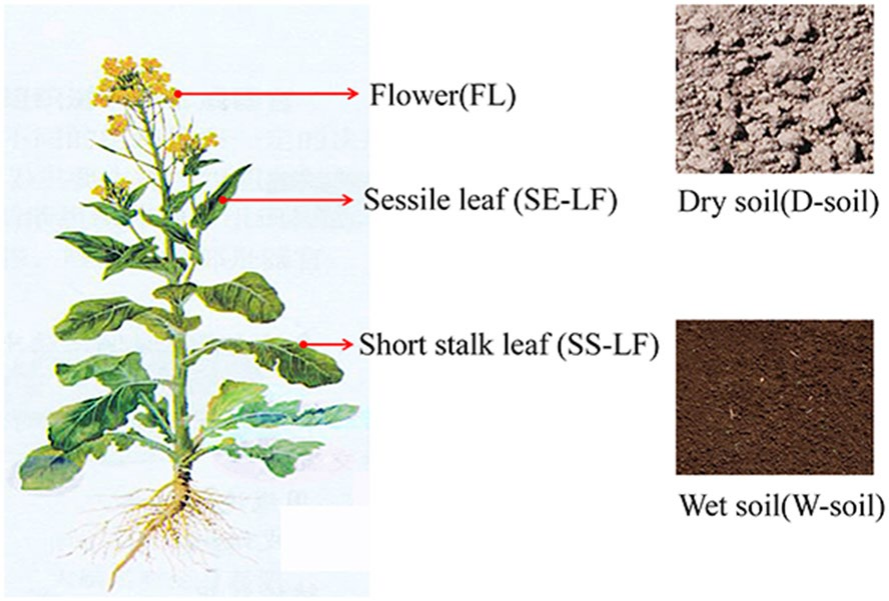
Endmembers selected in this study

To analyze the factor of spectral mixture within a pixel, five endmembers were considered in this study: (1) flower (FL), (2) sessile leaf (SE-LF), (3) short stalk leaf (SS-LF), (4) wet soil (W-soil) and (5) dry soil (D-soil). They were the dominant components visible in our studied scene (Fig. 2). Samples of each component were collected from the study area and their spectra were immediately measured in situ using a hyperspectral radiometer (Analytical Spectral Devices Inc., Boulder, CO, USA). This radiometer was equipped with a 25° field-of-view optical fiber that obtained sample reflectance in range of 350–1100 nm at a spectral resolution around 1 nm. The measurements of W- or D- soil spectra were conducted in all plots (at least six sampling areas per plot) with the ASD fiber pointing to the area at the appropriate height to make sure the instant field of view was all covered by wet or dry soil with no vegetation, and the averaged spectra was used as soil spectra. The leaf spectra were taken using ASD with a self-illuminated leaf clip for sessile leaf and short-stalk leaf respectively. For each leaf, spectral reflectance was scanned at 5 positions randomly distributed on the leaf adaxial side and six leaves were sampled per plot. The average of all spectra scans was then used as leaf reflectance. Since the rapeseed flower is small and narrow, the sample flowers were gathered together on a black background and arranged to fully cover the sensor’s view field to make sure that the radiometer collected the pure spectra of flower. By this way, the reference endmember reflectance of five components were obtained: $\rho_{FL}$, $\rho_{SE - LF}$, $\rho_{SS - LF}$, $\rho_{W - soil}$ and $\rho_{D - soil}$.

For spectral mixture analysis, the linear mixing spectral model [70] was used in this study to estimate the fractional abundance of each spectral endmember. It is assumed that the acquired image can be represented as a linear mixture of a few dominant spectral endmembers. For a given pixel at the wavelength λ, the pixel reflectance ρ(λ) can be approximated as:

3 $$\uprho\left(\uplambda \right) = \mathop \sum \limits_{i = 1}^{N} Abd_{i} \rho_{i} \left( \lambda \right);\quad {\text{and}}\quad 0 \le Abd_{i} \le 1;\quad {\text{and}}\quad \mathop \sum \limits_{i = 1}^{N} Abd_{i} = 1$$

where N is the number of selected endmembers, Abd_i_ is the fractional abundance of endmember i, $\rho_{i} \left( \lambda \right)$ is the reference reflectance of endmember i at band λ. The abundance is constrained between 0 and 1, and for each pixel the sum of the abundance of all endmembers equals to 1. An abundance of 0 indicates no spectral contributions from the particular endmember, while an abundance of 1 means this pixel spectra is the same with pure spectra of the particular endmember.

In this study, we selected flower, sessile leaf, short-stalk leaf, wet soil and dry soil as five endmembers. According to Eq. 3, abundance of the selected five components for each pixel can be retrieved from the six-band UAV image of the study site [71–73] (run by MATLAB 7.5) as:

4 $$\left[ {\begin{array}{*{20}c} {\rho (\lambda_{1} )} \\ {\rho (\lambda_{2} )} \\ {\rho (\lambda_{3} )} \\ \begin{aligned} \rho (\lambda_{4} ) \hfill \\ \rho (\lambda_{5} ) \hfill \\ \rho (\lambda_{6} ) \hfill \\ \end{aligned} \\ \end{array} } \right] = \left[ {\begin{array}{*{20}c} {\rho_{\text{FL}} (\lambda_{1} )} & {\rho_{\text{SE-LF}} (\lambda_{1} )} & {\rho_{\text{SS-LF}} (\lambda_{1} )} & {\rho_{\text{W-soil}} (\lambda_{1} )} & {\rho_{\text{D-soil}} (\lambda_{1} )} \\ {\rho_{\text{FL}} (\lambda_{2} )} & {\rho_{\text{SE-LF}} (\lambda_{2} )} & {\rho_{\text{SS-LF}} (\lambda_{2} )} & {\rho_{\text{W-soil}} (\lambda_{2} )} & {\rho_{\text{D-soil}} (\lambda_{2} )} \\ {\rho_{\text{FL}} (\lambda_{3} )} & {\rho_{\text{SE-LF}} (\lambda_{3} )} & {\rho_{\text{SS-LF}} (\lambda_{3} )} & {\rho_{\text{W-soil}} (\lambda_{3} )} & {\rho_{\text{D-soil}} (\lambda_{3} )} \\ {\rho_{\text{FL}} (\lambda_{4} )} & {\rho_{\text{SE-LF}} (\lambda_{4} )} & {\rho_{\text{SS-LF}} (\lambda_{4} )} & {\rho_{\text{W-soil}} (\lambda_{4} )} & {\rho_{\text{D-soil}} (\lambda_{4} )} \\ {\rho_{\text{FL}} (\lambda_{5} )} & {\rho_{\text{SE-LF}} (\lambda_{5} )} & {\rho_{\text{SS-LF}} (\lambda_{5} )} & {\rho_{\text{W-soil}} (\lambda_{5} )} & {\rho_{\text{D-soil}} (\lambda_{5} )} \\ {\rho_{\text{FL}} (\lambda_{6} )} & {\rho_{\text{SE-LF}} (\lambda_{6} )} & {\rho_{\text{SS-LF}} (\lambda_{6} )} & {\rho_{\text{W-soil}} (\lambda_{6} )} & {\rho_{\text{D-soil}} (\lambda_{6} )} \\ \end{array} } \right]\left[ {\begin{array}{*{20}c} {{\text{Abd}}_{\text{FL}} } \\ {{\text{Abd}}_{\text{SE-LF}} } \\ {{\text{Abd}}_{\text{SS-LF}} } \\ {{\text{Abd}}_{\text{W-soil}} } \\ {{\text{Abd}}_{\text{D-soil}} } \\ \end{array} } \right]$$

where $\uprho\left( {\lambda_{i} } \right)$ is the surface reflectance of the given pixel at band $\lambda_{i}$ (i = 1, 2…6). $\rho_{FL} \left( {\lambda_{i} } \right)$, $\rho_{{SE{-}LF}} \left( {\lambda_{i} } \right)$, $\rho_{{SS{-}LF}} \left( {\lambda_{i} } \right)$, $\rho_{{W{-}soil}} \left( {\lambda_{i} } \right)$ and $\rho_{{D{-}soil}} \left( {\lambda_{i} } \right)$ are the endmember reflectance at band $\lambda_{i}$ for flower, sessile leave, short stalk leave, wet soil and dry soil, respectively. $Abd_{FL}$, $Abd_{{SE{-}LF}}$, $Abd_{{SS{-}LF}}$, $Abd_{{W{-}soil}}$ and $Abd_{{D{-}soil}}$ are the abundance of flower, sessile leave, short stalk leave, wet soil and dry soil respectively, referring to the fraction of the given component within a pixel. Pixel by pixel, the abundance images of five endmembers were then constructed. For each abundance image, the previous defined rectangle in each of 24 plots for calculating plot-level VI was used to retrieve plot-level abundance by averaging abundance values of all pixels within the given rectangle.

### Yield estimation in rapeseed using VI and abundance data

In this study, plot-level VI was firstly correlated with rapeseed yield directly. Since leaves are the main organ for photosynthesis in rapeseed that will determine its production and the seed number largely depends on the number of flowers that will be further translated into pods, plot-level VI was multiplied by plot-level leaf or flower abundance for relating to rapeseed yield. As linear relationships are easy to implement and sensitive to wide range of variation in the dependent variable [74], four linear relationships were developed using 24 samples: (1) yield versus VI, (2) yield versus VI × *Abd*_*FL*_ (3) yield versus VI × **(**Abd_SE-LF_ + Abd_SS-LF_) and (4) yield versus VI × Abd_SS-LF_. Coefficients of determination (R^2^) and coefficients of variation (CV) were analyzed and compared.

### Algorithm establishment using leave-one-out cross-validation

This study used the leave-one-out cross-validation approach [75] to establish the algorithm for rapeseed yield estimation. The samples were trained and tested for K times (K is the number of samples, K = 24 in this study). For each time i, K − 1 samples were used iteratively as training data for calibrating the coefficients (Coef_i_) of the algorithm with the accuracy of the coefficients of determination (R_i_^2^), and the remaining single sample was used for validation to obtain the estimation error (E_i_). This procedure was repeated K times, with all samples used for both calibration and validation and each sample used exactly one time as validation data. From K iterations, the final algorithm with the accuracy (R^2^ and root mean square error—RMSE) can be produced as:

5 $$Coef = \frac{{\mathop \sum \nolimits_{i = 1}^{K} Coef_{i} }}{K} \quad R^{2} = \frac{{\mathop \sum \nolimits_{i = 1}^{K} R^{2}_{i} }}{K}\quad {\text{RMSE}} = \sqrt {\frac{{\mathop \sum \nolimits_{i = 1}^{K} E_{i}^{2} }}{K}}$$

## Results

### Relationship of VI versus yield in rapeseed

**Fig. 3:**
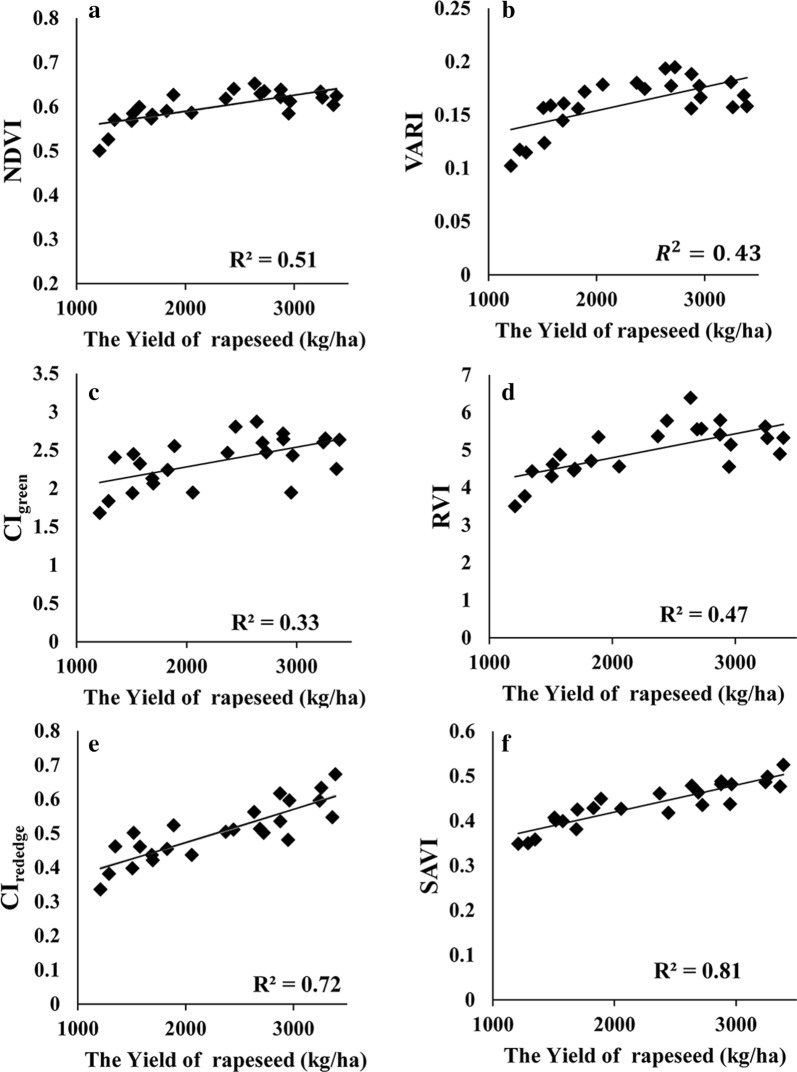
The relationships of yield and **a** CI_green_, **b** RVI, **c** NDVI, **d** VARI, **e** CI_red edge_ and **f** SAVI

In this study, the yield was firstly correlated with several widely used VIs. Among the tested indices, CI_red edge_, EVI, DVI, RDVI, TVI and SAVI showed significant correlations with yield (R^2^ > 0.7) in rapeseed, while NDVI, RVI, VARI and CI_green_ had weak correlations with rapeseed yield (R^2^ below 0.52)—Table 2. In addition, the relationships of NDVI and VARI versus yield appeared nonlinear. As shown in Fig. 3, NDVI and VARI were saturated to moderate to high yield variations when the yield of rapeseed exceeding 2000 (kg/ha), but SAVI and CI_red edge_ related to yield almost linearly.

**Table 2:** Correlation coefficient (R^2^) between VI and yield in rapeseed

	CI_green_	VARI	RVI	NDVI	CI_red edge_	EVI	DVI	RDVI	TVI	SAVI
R^2^	0.33	0.43	0.47	0.51	0.72	0.74	0.78	0.78	0.78	0.81

### Image-based abundance analysis

**Fig. 4:**
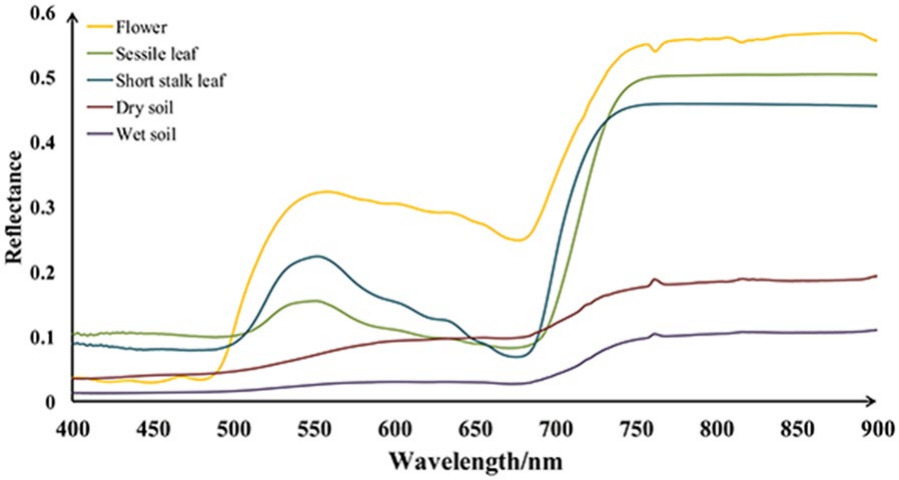
Pure spectral reflectance of flower, sessile leaf, short stalk leaf, dry soil and wet soil in the studied rapeseed plots

In order to improve the accuracy of yield estimates, spectral mixture was considered as a factor affecting yield in our developed approach. Figure 4 presented the measured spectra of five endmembers appearing in the studied rapeseed-plot. As soil moisture increased, soil reflectance decreased at all wavelengths. Obvious spectra difference was observed for flower, sessile leaf and short stalk leaf in rapeseed plant. Flower reflectance was lower than half of the leaf reflectance in blue band (3% vs. 8%), but it was much higher than leaf reflectance in green, red and NIR bands. Compared to short stalk leaf, sessile leaf had much lower green reflectance but a little higher NIR reflectance.

**Fig. 5:**
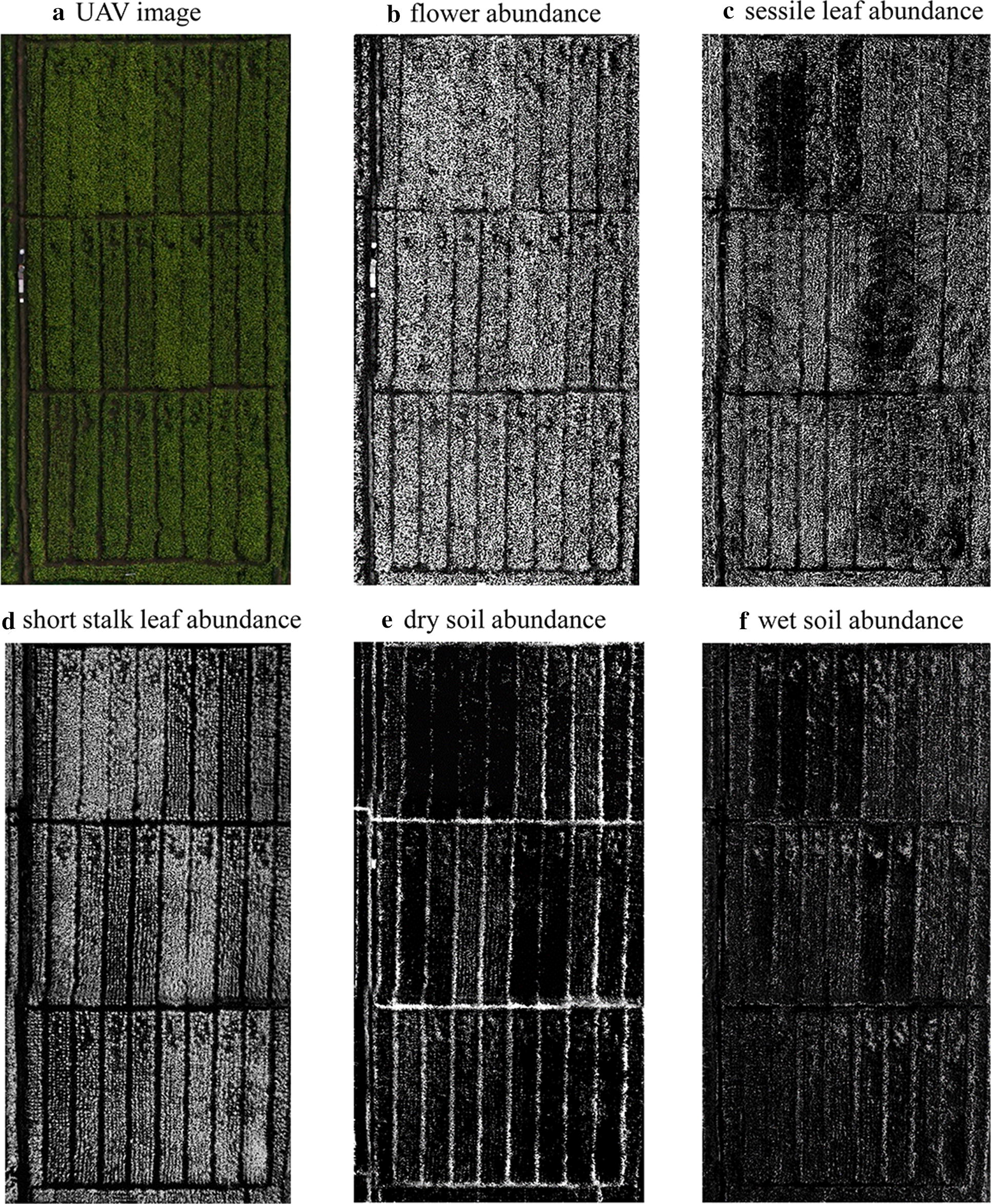
**a** The six-band image of the study area obtained by UAV system (true color was shown). Abundance images derived from spectral mixture analysis on the UAV six-band image for **b** flower, **c** sessile leaf, **d** short stalk leaf, **e** dry soil and **f** wet soil

Based on spectra of selected endmembers, abundance image of each component was derived for the study area. The abundance images of flower, short stalk leaf, sessile leaf, wet soil, and dry soil were given in Fig. 5. Generally, among the five abundance images, flower abundance image appeared the brightest. The abundance image of short stalk leaf was overall brighter than that of sessile leaf. And the brightness of dry and wet soil abundance images was relatively low (Fig. 5b–f). Pixels located at the ridges between the plots were bright in soil abundance images but dark in flower/leaf abundance images. Noted that obvious brightness heterogeneity was existed among different plots in the images, and such heterogeneity patterns were quite different in flower abundance image and leaf abundance images.

### Yield estimation using VI and abundance data

**Fig. 6:**
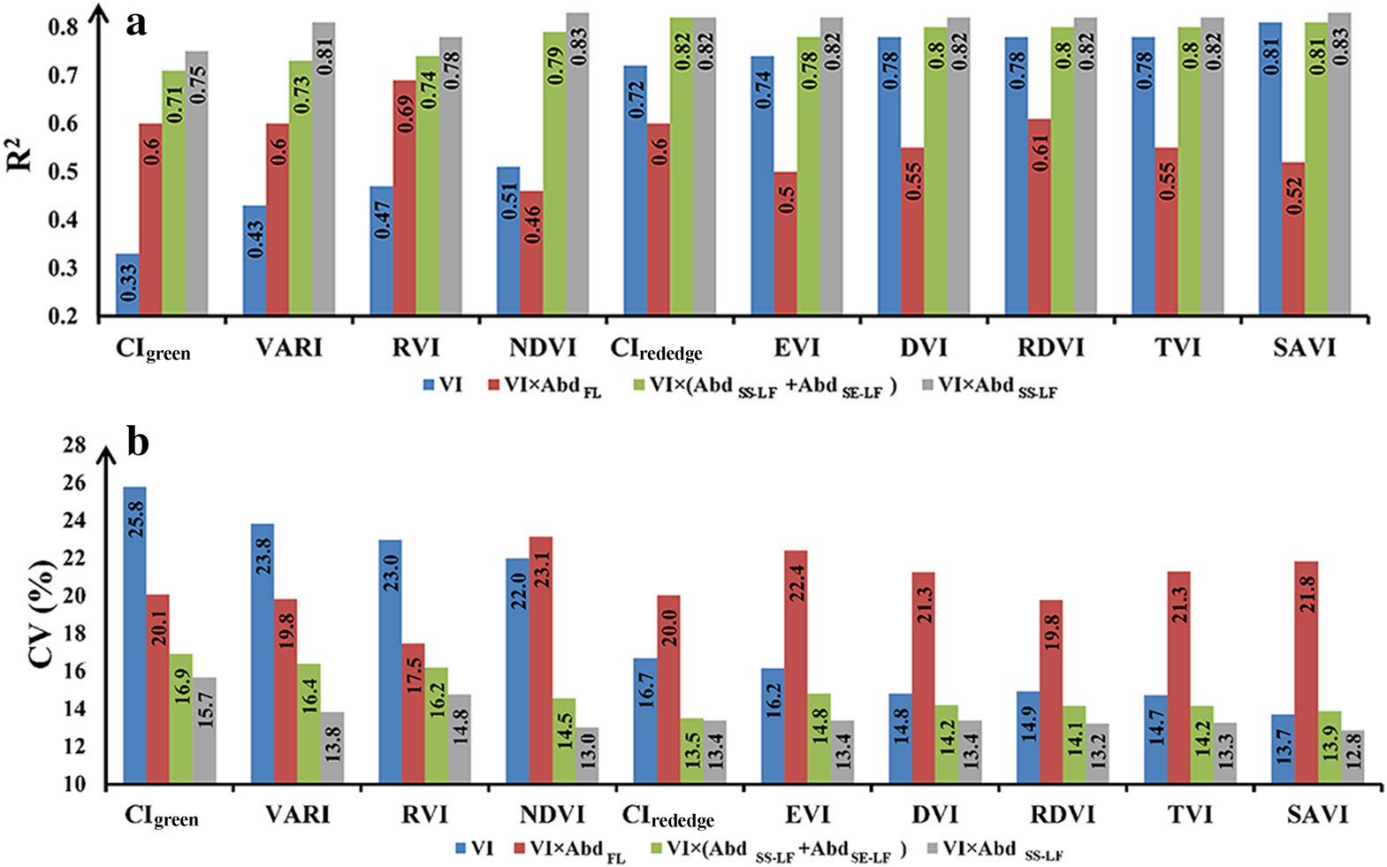
The comparison of **a** coefficients of determination (R^2^) and **b** coefficients of variation (CV) for relationships of (1) yield versus VI, (2) yield versus VI × *Abd*_*FL*_ (3) yield versus VI × (*Abd*_*SE*-*LF*_ + *Abd*_*SS*-*LF*_) and (4) yield versus VI × *Abd*_*SS*-*LF*_ for the studied indices

**Fig. 7:**
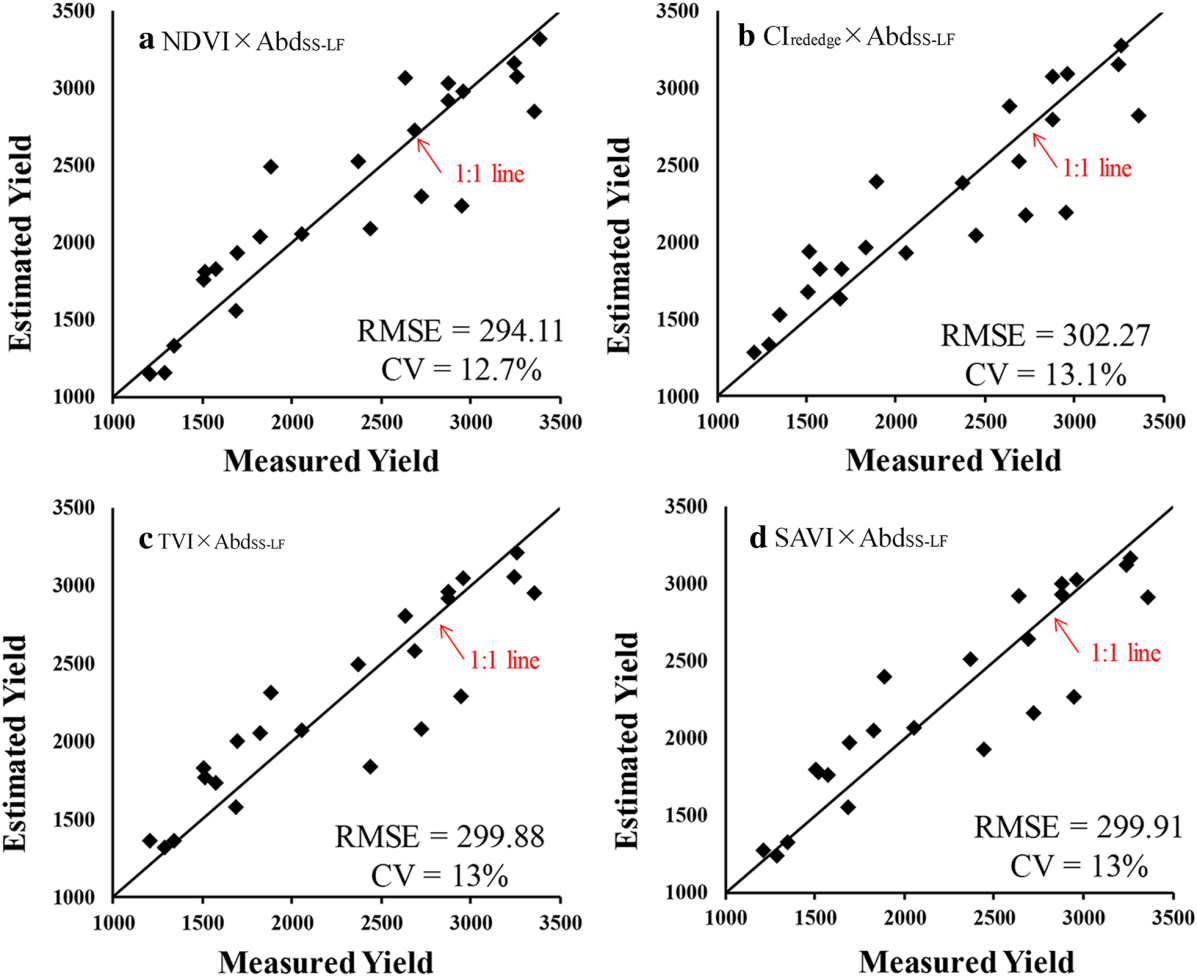
Validation of algorithms, established using the leave-one-out cross-validation approach, for estimating rapeseed yield in 24 plots under different nitrogen treatments by **a** NDVI × Abd_SS-LF_, **b** CI_red edge_ × Abd_SS-LF_, **c** TVI × Abd_SS-LF_ and **d** SAVI × Abd_SS-LF_

Since flower and leaf were the most important organs for rapeseed photosynthesis and production, in our proposed approach we used the information of plot-level flower abundance (Abd_FL_), leaf (sessile leaf and short stalk leaf together) abundance **(**Abd_SE-LF_ + Abd_SS-LF_) and short stalk leaf abundance (Abd_SS-LF_) to evaluate the yield in rapeseed. Generally, using VI × Abd_FL_ to estimate rapeseed yield was less accurate than using VI alone with higher CV and lower R^2^ values except for CI_green_, VARI and RVI. For all tested indices, multiplying leaf-related abundance information (VI × (Abd_SE-LF_ + Abd_SS-LF_) and VI × Abd_SS-LF_) increased the accuracy of yield estimation (Table 3). As shown in Fig. 6, using the product of leaf-related abundance and VI was able to estimate yield accurately with R^2^ above 0.7 and CV blow 17 (%). Especially for the indices which had weak correlation with yield (such as NDVI, CI_green_, VARI, RVI), the yield estimation accuracy was greatly improved when using VI × (Abd_SE-LF_ + Abd_SS-LF_) and VI × Abd_SS-LF_, with R^2^ increased by 0.3 and CV decreased by 8%. Also noticed, for all indices VI × Abd_SS-LF_ consistently gave better estimation results than VI × (Abd_SE-LF_ + Abd_SS-LF_). The algorithms were established using the leave-one-out cross-validation approach for NDVI × Abd_SS-LF_, CI_red edge_ × Abd_SS-LF_, TVI × Abd_SS-LF_ and SAVI × Abd_SS-LF_, which had the highest correlation with yield (Table 4). They worked accurately for estimating yield in rapeseed with RMSE below 303 kg/ha and CV below 13.1% (Fig. 7). Moreover, the relationship of yield versus NDVI × Abd_SS-LF_ appeared much more linear related to yield than the relationship of yield versus NDVI (Figs. 3 and 7).

**Table 3:** The coefficients of determination (R^2^) and coefficients of variation (CV) of relationships of yield versus VI, yield versus VI × Abd_FL_, yield versus VI × (Abd_SE-Lf_ + Abd_SS-LF_) and yield versus VI × Abd_SS-LF_

	*R* ^2^				CV (%)			
	VI	VI × Abd_FL_	VI × (Abd_SE-LF_ + Abd_SS-LF_)	VI × Abd_SS-LF_	VI	VI × Abd_FL_	VI × (Abd_SE-LF_ + Abd_SS-LF_)	VI × Abd_SS-LF_
CI_green_	0.33	0.6	0.71	0.75	25.8	20.1	16.9	15.7
VARI	0.43	0.6	0.73	0.81	23.8	19.8	16.4	13.8
RVI	0.47	0.69	0.74	0.78	23.0	17.5	16.2	14.8
NDVI	0.51	0.46	0.79	0.83	22.0	23.1	14.5	13.0
CI_red edge_	0.72	0.6	0.82	0.82	16.7	20.0	13.5	13.4
EVI	0.74	0.5	0.78	0.82	16.2	22.4	14.8	13.4
DVI	0.78	0.55	0.8	0.82	14.8	21.3	14.2	13.4
RDVI	0.78	0.61	0.8	0.82	14.9	19.8	14.1	13.2
TVI	0.78	0.55	0.8	0.82	14.7	21.3	14.2	13.3
SAVI	0.81	0.52	0.81	0.83	13.7	21.8	13.9	12.8

**Table 4:** The algorithms for estimating rapeseed yield using the product of vegetation index and short-stalk-leaf abundance

VI × Abd_SS_LF_	Best fit function	R^2^	RMSE (kg/ha)
SAVI × Abd_SS_LF_	Yield = 9252.9 × SAVI × Abd_SS_LF_ +519.28	0.84	299.91
NDVI × Abd_SS_LF_	Yield = 8059.7 × NDVI × Abd_SS_LF_ +204.22	0.83	294.11
CI_rededge_ × Abd_SS_LF_	Yield = 7011.2 × CI_rededge_ × Abd_SS_LF_ +735.09	0.82	302.27
TVI × Abd_SS_LF_	Yield = 181.15 × TVI × Abd_SS_LF_ +755.43	0.82	299.88

The best fit functions, determination coefficients (R^2^) and root mean square errors (RMSE) are given for four indices

## Discussion

The indices tested in this study was mostly originally developed for estimating vegetation greenness-related parameters such as chlorophyll content, leaf area index and vegetation fraction. It is found that crop greenness during mature growing stage was indicative to crop yield and some indices have been successfully used for yield estimation in maize and soybean [76]. However, they didn’t work accurately for yield estimates in rapeseed (Table 2, Fig. 3). Especially for indices using green reflectance (CI_green_ and VARI), the relationships of VI versus yield were weak with R^2^ below 0.43. This is consistent with finding from Sulik and Long [77] that the correlation between NDVI and yield was only 0.22 in spring canola during flowering seasons in Oregon, USA, thus they proposed a yellowness index which was linearly and strongly related to canola yield with the correlation coefficient around 0.76. Unlike grain crops (e.g., maize or soybean), rapeseed during early mature stage had conspicuous flowers which may occupy the top of canopy for more than 30 days. The flowers are numerous and aligned in racemes, and they appear bright yellow. In this case, canopy reflectance in green bands would be more affected by flower absorption and scattering. On the other hand, plot-level VI was calculated from mixed components including flower, leaf and soil. Each component contributed differently to rapeseed yield, so using VI alone for yield regression may introduce unexpected uncertainties. Thus the abundance images of each component were produced trying to associate VI with the component most relevant to rapeseed yield.

Among the five abundance images, flower abundance was the brightest (Fig. 5) indicating that flowers occupied the largest proportion in view of sensor. This is not surprising since the rapeseed was blooming in our studied period, and flowers were growing on the top of canopy thus easily being seen by the sensor. Noted that the abundance of short stalk leaf was generally higher than that of sessile leaf. In rapeseed plant, sessile leaf was quite small and vertically oriented (Fig. 2), thus it was likely to be hidden underneath the flower petals. Although short stalk leaf was developed underneath the sessile leaf, it was much bigger and horizontal expanded thus appearing more visible in view of sensor. Due to the different nitrogen treatments applied in 24 plots, the greenness of rapeseed in different plots varied when the images were taken, and different plots would have contrasting yield thereafter ranging from 1000 to 3500 kg/ha. It is observed that flower abundance image was quite homogeneous in 24 plots, but obvious difference in leaf abundance existed among different plots (Fig. 5). It indicated that leaf abundance was more sensitive than flower abundance to nitrogen usage variations.

Compared to using VI to estimate yield, the accuracy of yield estimation increased when using VI × Abd_SS-LF_ and VI × (Abd_SE-LF_ + Abd_SS-LF_) for all indices, but for most indices the accuracy decreased when using VI × Abd_FL_ (Fig. 6, Table 3). The flowering period of rapeseed can last for more than 30 days. The plants begin to flower firstly at the main stems, and then on upper branches followed by lower branches. The studied image was taken in the early flowering period, thus the flowers that may bloom later were missing at the observation moment. Only one observation (even several) during the relatively long flowering period cannot record the complete information of possible flowers of all plants. So multiplying the flower abundance at one moment weakened the relationship of VI versus yield.

During flowering period, plant leaves were fully developed and their greenness maintained quite stable. Many studies showed that leaves of rapeseed are mainly responsible for photosynthesis which is crucial to final yield, and the leaf status at plant mature stage is representative to crop potential yield [78]. The product of VI and leaf-related abundance may somewhat get rid of components that are not closely related to rapeseed yield (e.g., soil and flower at one moment). For all tested VIs, VI × (Abd_SE-LF_ + Abd_SS-LF_) related to rapeseed yield closely with R^2^ above 0.7. Moreover, multiplying the abundance of short stalk leaf further increased the accuracy of yield for all indices. Wang et al.’s [35] experiments evaluated and compared the contributions of short stalk leaves and sessile leaves on rapeseed yield. They found that the removal of sessile leaves obviously decreased the number of rapeseed pods while the removal of short stalk leaves not only decreased the number of pods but also the number of seeds per pod. As shown in Fig. 6, by multiplying Abd_SS-LF_ all indices were able to estimate yield quite accurately with R^2^ above 0.75 and CV below 15.7%. Even for VIs appeared weakly related to rapeseed yield such as CI_green_, VARI and NDVI, the use of Abd_SS-LF_ enabled them to achieve comparable accuracy with other indices. VI × Abd_SS-LF_ associated VI to the fraction of short stalk leaf in a plot, which is the most relevant component for rapeseed yield, thus resulting in higher accuracy for yield estimation than VI alone. It indicated that the model of yield∝VI × Abd_SS-LF_ may be applicable for all greenness-related VIs and not selective to indices with specific spectral bands and sophisticated formulations, which greatly expand the range of choice for yield estimation using remotely sensed images with conventional and few bands.

This study developed an approach to estimate rapeseed yield using the product of vegetation index and leaf abundance retrieved from the UAV image. The approach is simple but gives an important indication that spectral mixture analysis needs to be considered when estimating yield by remotely sensed VI, especially for the image containing obviously spectral different components. For all tested VIs in rapeseed, the product of VI and leaf abundance was capable of estimating yield especially for those VIs which seemed weakly related to rapeseed yield in many studies [79, 80]. Instead of creating a new spectral index requiring specific spectral bands or sophisticated formulations, maybe an effective and simple way is to relate VI to abundance of plant components most relevant with its final yield. The results of this work can provide a conceptual background for using satellite data of which the spectral mixture may be an issue. The endmembers proposed in this study are particularly for rapeseed yield estimation, which is not applicable to other crops. But this work may offer a theoretical framework for yield estimation in crops which have conspicuous flowers or fruits with significantly different spectra from their leaves (e.g., rapeseed, cotton). Our future work is to apply this approach to real satellite data and in other crop species. In addition, we’d like to test this approach in crops planted in various regions under different weather conditions in order to explore the robustness of our approach to changes in meteorological parameters such as temperature, humidity, precipitation and wind speed.

## Conclusions

In this study, we developed an approach to estimate rapeseed yield using UAV-obtained canopy reflectance and abundance data. It is observed that canopy reflectance collected during rapeseed flowering period is mixed and confounded by reflectance of flower, leaf and soil. Thus, the spectral mixture analysis was conducted to estimate the fractional abundance of different components that appear in the studied scene within a pixel. Flower, sessile leaf, short stalk leaf, wet soil and dry soil were selected as endmemebers and abundance images of these components were produced based on the six-band UAV image. For all tested indices, the product of plot-level VI and leaf-related abundance closely related to rapeseed yield with R^2^ above 0.75. Among the tested VIs, multiplying NDVI, CI_red edge_, TVI, and SAVI by short-stalk-leaf abundance were the most accurate for yield estimates in rapeseed under different nitrogen fertilizer treatment with estimation errors below 13.1%.
